# Children’s Environmental Health in South and Southeast Asia: Networking for Better Child Health Outcomes

**DOI:** 10.5334/aogh.2403

**Published:** 2019-02-25

**Authors:** Peter D. Sly, Brittany Trottier, David Carpenter, Ubon Cha’on, Stephania Cormier, Betsy Galluzzo, Samayita Ghosh, Fiona Goldizen, Michelle Heacock, Paul Jagals, Hari Datt Joshi, Prachi Kathuria, Le Thai Ha, Melina S. Magsumbol, Panida Navasumrit, Poornima Prabhakaran, Banalata Sen, Chris Skelly, Inoka Suraweera, Sathiarany Vong, Chador Wangdi, William A. Suk

**Affiliations:** 1 Children’s Health and Environment Program, the University of Queensland, Brisbane, QLD, AU; 2 National Institute of Environmental Health Sciences, Research Triangle Park, NC, US; 3 Institute for Health and the Environment, University at Albany, Albany, NY, US; 4 Khon Kaen University, Khon Kaen, TH; 5 Louisana State University, Baton Rouge, LA, US; 6 MDB, Inc., Washington D.C., US; 7 Public Health Foundation of India, New Delhi, IN; 8 Yeti Health Science Academy, Purbanchal University, Kathmandu, NP; 9 Hriday Shan, Delhi, IN; 10 National Institute of Occupational and Environmental Health, VN; 11 Chulabhorn Research Institute, Bangkok, TH; 12 Public Health Dorset, Dorchester, UK; 13 Directorate of Environmental and Occupational Health, Ministry of Health, Colombo, LI; 14 Department of Preventive Medicine, Ministry of Health, KH; 15 Ministry of Health, Thimphu, BT

## Abstract

Children are particularly vulnerable to environmental hazards because they receive higher doses of pollutants in any given environment and often do not have equitable access to social protection mechanisms such as environmental and health care services. The World Health Organization established a global network of collaborating centres that address children’s environmental health (CEH). The network developed a focus on low- and middle-income countries (LMICs) and is broadening its reach by conducting regional workshops for CEH.

**Objective:**

This paper reports on the outcomes of a workshop held in conjunction with the 17th International Conference (November 2017) of the Pacific Basin Consortium for Environment and Health, focused on the state of CEH in South and Southeast Asia as presented by seven countries from the region (India, Bangladesh, Nepal, Bhutan, Vietnam, Thailand, Sri Lanka).

**Workshop outcomes:**

Country reports presented at the meeting show a high degree of similarity with respect to the issues threatening the health of children. The most common problems are outdoor and household air pollution in addition to exposure to heavy metals, industrial chemicals, and pesticides. Many children still do not have adequate access to clean water and improved sanitation while infectious diseases remain a problem, especially for children living in poverty. Child labour is widely prevalent, generally without adequate training or personal protective equipment. The children now face the dual burden of undernutrition and stunting on the one hand and overnutrition and obesity on the other.

**Conclusion:**

It is evident that some countries in these regions are doing better than others in varying areas of CEH. By establishing and participating in regional networks, countries can learn from each other and harmonise their efforts to protect CEH so that all can benefit from closer interactions.

## Introduction

Children are one of the most vulnerable groups within our society. They are exposed to higher doses of pollutants in any given environment, including low-level exposures occurring during fetal development and in early postnatal life that increase the lifelong risk of chronic disease. In many circumstances, children do not have equitable access to health care services or social protection mechanisms [1,2,3].

As highlighted by the recent Lancet Commission on Pollution and Health [3], pollution was responsible for 16% of global deaths in 2015, or approximately 9 million, which was three times more than deaths from AIDS, tuberculosis, and malaria combined. These deaths were not evenly distributed, with around 92% occurring in low- and middle-income countries (LMICs). Children are among the most vulnerable members of the population for both death and disability. Even low-level exposures occurring during fetal development and in early postnatal life increase the lifelong risk of chronic disease [3].

The World Health Organization (WHO) Department of Public Health, Environment, and Social Determinants of Disease is improving awareness of the health consequences of pollution and other adverse environmental exposures by establishing a series of WHO Collaborating Centres (WHOCCs) that work on various aspects of children’s environmental health (CEH). These centres have formed a network [4] coordinated by the WHOCC for Environmental Health Sciences, located at the National Institute of Environmental Health Sciences in Research Triangle Park, North Carolina, United States. The network has been active in promoting awareness of CEH through publications [1,5,6] and has a focus on in low- and middle-income countries (LMICs) [7,8,9].

The Network of WHO collaborating centres for CEH proactively broadened its reach beyond formal WHOCCs by holding regional workshops [8], with the most recent workshop held in conjunction with the 17th International Conference of the Pacific Basin Consortium for Environment and Health in New Delhi in November 2017. The purpose of the workshop was to gauge the state of CEH in South and Southeast Asian countries. Child populations in these countries vary considerably in size and also contribute substantially to the burden of disease in terms of mortality and disability-adjusted life years (Table 1). Delegates from several of these countries (India, Bangladesh, Nepal, Bhutan, Vietnam, Thailand, and Sri Lanka) attended the workshop and included representatives from government ministries, academia, national institutes of occupational and environmental health and sciences, and nongovernmental organizations. This paper summarises the reports presented at this workshop by the represented countries.

**Table 1:** Contribution of Children to the Burden of Disease in Terms of Mortality and Disability-Adjusted Life Years (DALYs) in Countries in South and Southeast Asia, 2016.

Country	Total population			Children under 5 years			Children 5–14 years		

	Number^1^	Mortality^*2^	DALYs^+2^	Number^3^	Mortality^*4^	DALYs^+2^	Number^3^	Mortality^*5^	DALYs^+2^

Afghanistan	33,369,944	13.4	18,559,913.78	4,925,453	70.4	6,668,140.81	9,529,049	9.5	1,563,032.74
Bangladesh	157,826,578	5.4	44,604,974.52	15,348,064	34.2	8,927,010.09	31,784,370	4.9	3,112,784.50
Bhutan	758,288	6.5	217,940.45	64,238	32.4	48,462.11	142,017	7.1	15,665.39
Cambodia	16,204,486	7.5	5,151,857.17	1,769,672	30.6	1,156,240.29	3,199,351	5.3	317,402.72
India	1,281,935,911	7.3	466,336,532.09	122,905,717	43	82,178,860.46	253,480,869	6.3	28,678,894.59
Indonesia	260,580,739	6.5	72,732,990.22	25,390,959	26.4	10,578,185.18	46,353,414	5.3	3,829,692.22
Laos	7,126,706	7.4	2,924,169.05	837,524	63.9	1,325,941.62	1,543,987	9.9	175,417.86
Malaysia	31,381,992	5.1	6,737,516.32	2,565,658	8.3	355,847.46	4,877,968	2.6	313,839.04
Maldives	392,709	4.0	60,862.34	37,880	8.5	5,214.37	63,593	2.5	4,293.56
Mauritius	1,277,459	7.1	381,231.49	70,171	13.7	18,854.55	170,552	1.8	12,091.07
Myanmar	55,123,814	7.4	17,015,425.21	4,510,575	50.8	2,442,508.47	10,166,907	8.2	904,869.13
Pakistan	204,924,861	6.3	70,625,422.15	24,938,283	78.8	23,090,427.29	42,325,803	11.3	5,107,578.01
Philippines	104,256,076	6.1	30,068,249.49	11,363,634	27.1	5,388,348.70	21,048,184	6.5	2,130,934.32
Seychelles	97,026	7.0	27,257.19	8,299	14.3	1,879.32	14,597	3.8	958.53
Sri Lanka	22,409,381	6.2	5,000,512.32	1,602,492	9.4	220,579.92	3,450,933	2.3	236,149.00
Thailand	68,414,135	8.0	18,341,441.31	3,739,823	12.2	417,806.67	8,136,696	3.2	536,483.62
Timor-Leste	1,211,244	5.9	322,385.06	217,743	49.7	108,399.07	300,858	8.0	28,388.64
Vietnam	96,160,163	5.9	24,140,772.25	7,751,809	21.6	1,928,964.37	14,042,712	2.8	872,845.00

* Per 1,000 live births. ^+^ All causes, all sexes. ^1^ The World Factbook. Washington D.C.: Central Intelligence Agency. https://www.cia.gov/library/publications/the-world-factbook/index.html. Accessed July 12, 2018. ^2^ GBD Compare Data Visualization. Seattle, WA: Institute for Health Metrics and Evaluation, University of Washington; 2017. https://vizhub.healthdata.org/gbd-compare. Accessed July 12, 2018. ^3^ Population Pyramids of the World from 1950 to 2100. https://www.populationpyramid.net. Accessed July 12, 2018. ^4^ Global Health Observatory Data Repository. Probability of dying per 1000 live births: Data by country. Geneva: World Health Organization; 2017. http://apps.who.int/gho/data/view.main.182?lang=en. Updated September 19, 2017. Accessed July 12, 2018. ^5^ Global Health Observatory Data Repository. Probability of dying per 1000 children, aged 5 to 14: Data by country. Geneva: World Health Organization; 2017. http://apps.who.int/gho/data/node.main.CM5TO14?lang=en. Updated September 21, 2017. Accessed July 12, 2018.

## CEH in India

There are a number of health threats to children in India related to the environment. Indoor and outdoor air pollution from industries, traffic, and biomass fuel burning increase exposures. Small-scale industries emit dangerous nanoparticles from their processes. Electricity generators, including those used domestically, release diesel exhaust particulates. Children also constitute a substantial section of the informal labour workforce in Indian cities, engaging in garbage collection, segregation and disposal, electronic waste recycling, and several other occupations that expose them to hazardous environmental pollutants. Pollutants in water bodies, often the only source of drinking water for many sections of society in India, are major sources of waterborne diseases.

Other sources of environmental pollutants that Indian children are exposed to are landfill sites. Families from lower socioeconomic strata live on these sites and earn a living from rummaging around the landfills all day and picking out waste that have financial value. Whole families collect waste together, in poor living conditions near or on dumping sites that have toxins, chemicals, and other hazards. There is an added social dynamic where children and families move as large groups and are territorial about their areas of collection. Working on these landfill sites, children—in utero or in postnatal life—are exposed to a toxic cocktail of chemicals due to lack of personal protection equipment, such as overall clothes, gloves, masks, goggles, and footwear. This results in eye, skin, and finger-prick injuries; respiratory and gastrointestinal disorders; and often poor intellectual and cognitive development. The National Institute of Occupational Health (NIOH) is conducting a study of “rag pickers” in Bangalore as part of their effort to collect exposure and health outcome data on children in the informal waste disposal and recycling sector. Some Indian studies have reported that these children have poor hygiene and nutrition, are exposed to waste materials, and commonly have leg injuries, gastrointestinal infections, worm infestation, scabies, skin diseases, pediculosis, and rabies (because of stray dogs) [10,11]. Other research has found polychlorinated dibenzodioxins (PCDDs) and polychlorinated dibenzofurans (PCDFs) at dumping sites at levels that exceed guidelines [12]. The risk of cancer, particularly due to polycyclic aromatic hydrocarbons (PAHs) and other carcinogens and leachate from landfills, is high [13,14]. An increased risk of tuberculosis, bronchitis, asthma, and pneumonia has been reported [11].

Children in rural areas often work in agriculture, frequently as unpaid labor for their own family. Pesticide exposure, particularly when it is prepared as well as used within the home, is a major health risk [15]. Children also work in supposedly nonhazardous roles, such as picking flowers. This requires that children be in fields before they attend school later in the day. This sometimes coincides with early morning pesticide applications and thus exposures. Children often don’t use protective apparel and therefore have dermal pesticide exposure, which is compounded by taking their food with them and not washing hands before eating. The resultant exposure can cause neurological disorders, even at low doses [16].

Another NIOH study looking at sheep farming reported on two more exposures. Sheep wool (used for carpets) expose nearby children through inhalation of dust and proteins from sheep wool. The study team also found dichlorodiphenyltrichloroethane (DDT) and other pesticides on the persons of the farming families, including their children.

Brick kilns remain a major health and occupational safety challenge in India in terms of emissions and heat stressors associated with these industries [17,18,19]. The government banned child labour in the brick industry, except for children working with their family in the industry. Children also work in extremely dangerous stone quarries [20], or parents have to bring the children to work with them, exposing nonworking children to high levels of silica dust in the vicinity of the quarries [21]. Mining and quarries are very noisy areas, and noise-induced hearing loss is common, even in children.

## CEH in Bangladesh

Air pollution is a serious issue in Bangladesh. It gets the most attention in the major cities, but rural air pollution remains poorly understood and might also be a major problem, especially for mothers and children who are impacted the most by indoor air pollution [22,23].

The collection and disposal of waste in Bangladesh presents a major problem for the population and the municipal authorities [24]. Collected waste is disposed mostly in landfills. Child waste pickers are often at the landfills, with significant adverse health outcomes common [25,26].

Lead poisoning from lead paint remains a major issue in many parts of the world, including Asian countries [27]. Elevated blood lead levels is a problem for children in Bangladesh [28,29]. In 2012, seven different organizations in Asia started Switchasia [30], a lead-free paint promotion project with Bangladesh, Nepal, Sri Lanka, the Philippines, India, and Indonesia being part of the project. Many manufacturers in these countries have reduced the lead content and are moving to zero lead-containing paint. Regulating the content of paint to reduce exposure is commendable, but enforcement remains a problem. Moreover, the issue of “legacy lead” in the environment will remain a problem unless adequate remediation is undertaken [27].

Toxic toys are an underreported issue but are of increasing recognition and importance [31,32]. Tests of toys sold in Bangladesh have found it to contain lead, cadmium, bromine, and chromium—some at levels more than 97% above the EU ceiling [33]. In one test, a Rubik’s cube from Bangladesh was found to have OctaBDE, a chemical banned under the Stockholm Convention. Most of the unsafe toys are produced in China, Thailand, and India. The products are spreading all over Asia, beyond countries where the toys are produced. Children’s cosmetics and toy jewellery may also have elevated levels of harmful substances. The Environment and Social Development Organization found arsenic in baby lotion and titanium dioxide in children’s jewellery [33].

Diarrhea, one of the leading causes of death in children younger than five years, accounted for 9% of 5.8 million global deaths and 6% of 0.119 million deaths in Bangladesh in 2015 [34].

The occupational hazards of child labour are another major issue in Bangladesh [35]. For instance, children who work in tanneries are exposed to heavy metals (lead, cadmium, mercury) [36]. Bangladesh has 50,000 children working in informal electronic waste (e-waste) recycling. Shipbreaking is a major industry in Bangladesh, but the shipbreaking yard is a “killing field.” Many children working there are exposed to toxic heavy metals from e-waste and scrap materials from the ships [37,38].

## CEH in Nepal

Indoor and outdoor air pollution are major risks for children [3]. Use of solid and biomass fuel is a problem in Nepal; 64% of indoor cooking is done with firewood, and 10% of households burn cow dung. This is particularly problematic in the mountainous areas of the country, where there is limited or no ventilation in the home due to cold outdoor temperatures [39], resulting in prolonged exposure to high levels of PM_10_ and PM_2.5_. These exposures result in increased rates of eye and respiratory ailments [40]. Ambient air pollution, particularly in Kathmandu, is a major challenge and problem [41]. Pollution comes from brick laying, construction projects, and vehicles. The pollution is so hazardous that a government campaign encourages people not to walk in the mornings in the Kathmandu Valley. This pollution is particularly a threat to children walking to school in the mornings.

Water, sanitation, and hygiene (WASH) challenges are major threats to CEH. Although 48% of households have safe drinking water, 38% have unimproved sanitation, and 15% practice open defecation or have no sanitation facility. There is limited systemic monitoring of water quality, and many studies have found varying levels of contaminants, making the water unsafe to use [42]. In schools, government policy requires one toilet for every 50 students, however the reality is that there are, on average, 127 students per toilet. Lack of toilet facilities has been shown to reduce female school attendance and therefore, female literacy. There is a correlation between toilets in schools and female literacy across Nepal.

Additional exposures and risks to children in Nepal include child labour in brick laying and similar industries, lead pollution, and climate change [43]. All five major vector-borne diseases are now endemic in Nepal, and there has been an observed shift in the range of vectors to more than 2,000 meters above sea level. Cold waves are a new issue in Nepal, with an observed 5% increase in the incidence of acute respiratory illnesses (ARI) in cold months. Deaths from ARI have increased by 2.68% for every one degree Celsius decrease in the daily minimum temperature.

In terms of CEH policies in Nepal, health is noted in the 2016 constitution. The National Health Policy of 2014 and National Water Supply and Sanitation Policy of 2014 are relevant to CEH issues. Nepal has had a climate change policy since 2011, and national improvement plans for WASH and for the health sector have been established. Implementation of most policies is overseen by the Ministry of Health, however child welfare is governed by the Ministry of Women, Children, and Social Welfare.

The challenges to improving CEH in Nepal are mainly a lack of coordination among government organizations and a lack of, or weak, implementation and enforcement of existing policies. Some health interventions require a change in cultural practices and behavior, such as the relationship between preferred household design and indoor cookstove use. Geographic challenges make delivery of care difficult and compound the complexity of disasters, deforestation, and climate change.

## CEH in Bhutan

The total population of Bhutan is less than 1 million people. Seventy percent of the country is forested, with a constitutional mandate to maintain a 60% forest cover; 58% of the population is dependent on agriculture; 99.5% of households have access to improved drinking water; and 92% have access to improved sanitation [44]. The leading causes of disease in children under five years in Bhutan are ARI, skin diseases, diarrhea, and digestive system diseases.

Although population access to electricity is very high, air pollution-related diseases remain in the top 10 diseases among children [44]. While 95% of the population has access to electricity for cooking, around 20% uses solid fuels for cooking. There is a preference for wood-style cooking for the flavor imparted to the food. This is particularly common in rural areas, where solid fuel is used by more than 33% of the population. In many of these homes, women carry the children on their back when cooking, thus increasing cooking-related indoor air exposures. Wood combustion for winter heating is used in traditional homes and in rural areas. Some urban areas continue to use traditional stoves and kerosene heaters during winter.

Drinking water and sanitation coverage has improved throughout the country, however diarrhea and waterborne diseases remain a leading cause of under-five mortality and morbidity. A preference for open defecation remains in some areas, despite the availability of improved sanitation facilities.

The health of children in Bhutan has been steadily improving [45,46] Stunting in children has decreased significantly but remains in rural areas and the eastern region of the country. Anemia remains common in children under five in Bhutan. Although rates have declined, more than 43% of children are anemic. Pregnant women have good health coverage, with many attending prenatal visits and with anemia rates lower than those of nonpregnant women, however only 52% of pregnant women receive prenatal care in the first trimester. Breastfeeding rates are high, with nearly half of women reporting exclusive breastfeeding. Upon weaning from breastfeeding, there is reported low dietary diversity for introduced complementary foods, with a low percentage of children being given iron-rich foods at ages 6–23 months. The Ministry of Health is seeking to distribute nutrition powder to health centres, particularly where iron-rich foods are less available.

Waste generation and disposal is an emerging problem, especially in urban areas. There is no separation of waste in Bhutan [47]. Informal waste collectors sort and resell scraps from waste. The capital, Thimphu, generates nearly 50 tons of waste per day, which is disposed of into one landfill 12 kilometers from the city. None of the wastes are segregated, sorted, or recycled. Leaching of chemicals and fires (accidental and intentional) at landfills contribute to water and air pollution, respectively.

Very few chemicals are manufactured in the country, with most products imported from India. Pesticides are used often, and asbestos is commonly used in many areas. Legislation to deal with carcinogenic chemicals is nonexistent, with no regular monitoring or awareness of carcinogens.

## CEH in Vietnam

There are increasing health disparities between the Kinh majority and ethnic minorities, between urban and rural residents, and between those in mountainous areas of the country compared to the lower delta areas [48,49]. Forty percent of poor children live in rural areas, with child poverty especially high in the northern mountains. Approximately 50% of rural children attend preschool, whereas 75% of urban children do.

The under-5 mortality rate in Vietnam is 20.2 per 1,000 live births, and diarrhea is the leading cause of under-5 death [50]. Additional diseases commonly impacting children include dengue fever and hand, foot, and mouth disease. A high proportion of Vietnamese children do not have access to clean drinking water, reaching upward of 80% in the Highlands and Mekong River Delta. More than half (53%) of schools don’t provide drinking water for students during school hours. Although 73% of schools have latrines, more than 50% are estimated to not meet adequate sanitation standards [51,52].

Neglected tropical diseases, including soil transmitted helminthiasis (STH), are a major threat to children’s health. Sixty-seven million people live in STH-endemic areas, and the most at risk are school-age children. In some areas, infection is as high as 86% of the population. Leading contributors to these high rates are lack of adequate sanitation, use of composted human waste in agriculture, barefoot walking, and consumption of raw vegetables [53,54].

Lead poisoning in children remains a problem in Vietnam [55]. Sources of lead exposure include lead mines, industrial production, recycling “villages” (where a majority of their industries are those that dismantle lead batteries), the use of traditional drugs which can include lead, and lead paint in toys. Lead battery recycling used to be common in residential areas, however new policies have moved activities to an industrial zone. A study in the recycling village of Dong Mai found blood levels of children in excess of 45 ug/dL (which usually is treated with chelation) [56]. After an intervention in the village, no children were found to have levels in excess of 45 ug/dL and the average level was reduced to under 15 ug/dL. Children’s blood levels have also been found to be elevated near lead mines in the country. There is a continued need to reduce lead poisoning in children. In doing so, efforts must include education, worker protection, and continued monitoring of interventions. Research is still needed in villages with issues similar to those in Dong Mai to explore preventative measures that may prevent children’s exposure.

Chronic arsenic contamination is common in many provinces and the main source of exposure is from contaminated groundwater [57]. There continues to be a need for sanitation and water in remote areas, but efforts to provide water from groundwater sources must address natural arsenic contamination as well, which makes the provision more difficult. A study on the effects of arsenic exposure on physical development, mental health in children, and genetic polymorphisms related to arsenic metabolism would be welcomed [58,59,60].

## CEH in Thailand

Neonatal and child mortality is low in Thailand, with a neonatal mortality rate of 3.5 per 1,000 live births and an under-5 mortality rate of 8.6 per 1,000 live births. Eight percent of live births are diagnosed with congenital anomalies, with the five most common birth defects in Thailand are congenital heart defects, limb abnormalities, cleft lip and palate, Down syndrome, and congenital hydrocephalus [61].

Thai women don’t breastfeed at the rates of many other neighboring countries, and they often breastfeed for a shorter duration of time [62]. An estimated 16% of children are stunted [63], yet Thailand has the fastest increasing rate for childhood obesity in the world [64]. There is a high prevalence of junk food consumed (food high in calories and fat), and soft drinks and sugary coffee are common in children’s diets.

Pesticides are commonly used on fruits and vegetables because growers like to protect crops from insects. Glyphosate and paraquat are commonly used in agriculture and have been found in high levels in maternal and foetal serum [65].

Flooding is a major threat to children’s health. In addition to threats usually associated with floods, schools often don’t have clean water after floods and thus, if open, are not safe for children. Chronic kidney disease of unknown aetiology (CKDu) is a growing problem, and the relationship between CKDu and exposures in water is being investigated [66].

Children’s exposure to toxic chemicals is a public health concern because children are one of the most susceptible groups in the population for exposure to environmental toxicants. The Chulabhorn Research Institute (CRI) has conducted research on children’s health impacts of exposure to environmental pollutants, such as traffic-related air pollutants, e-waste, and in utero exposure to arsenic. The potential health effects of urban air pollution related to traffic, particularly carcinogenic compounds including benzene, 1,3 butadiene, and PAHs, on children aged 9–13 years old was investigated through the use of various biomarkers. CRI studies clearly showed that children in Bangkok who were concurrently exposed to significantly higher levels of benzene, 1,3-butadiene, and PAHs had significantly higher levels of DNA damage, observed as elevated levels of 8-hydroxydeoxyguanosine (8-OHdG) and DNA strand breaks, and significantly lower DNA repair capacity compared to those of rural children [67,68].

The developing foetus is extremely vulnerable to effects of chemicals when exposure occurs in utero. A study in a Thai cohort has shown that arsenic exposure in utero increased expression of genes involved in various biological networks such as apoptosis, stress responses, and inflammation [69], and DNA damage in newborns, observed as increased levels of urinary 8-nitroguanine, which significantly correlated with increased expression of inflammatory genes (COX2, EGR1, and SOCS3) in cord blood [60]. A follow-up study in these prenatally arsenic-exposed children showed an increase in oxidative and nitrative DNA damage, represented by increased levels of 8-OHdG and 8-nitroguanine [60,70], as well as decreased expression of human 8-oxoguanine DNA glycosylase 1 (hOGG1), suggesting a defect in the repair of 8-OHdG.

Taken together, these results suggest that individuals with prenatal to early childhood exposure to environmental carcinogens are at a higher risk for developing disease and cancer later in life. The knowledge gained from these studies will lead to the establishment of national policies for the protection of health and the minimization of children’s health risk.

## CEH in Sri Lanka

The total midyear population of Sri Lanka is 21.4 million people [71], and 77% of children live in rural areas [72]. The under-5 mortality rate is 10 per 1,000 live births and the infant mortality rate is 8 per 1,000 live births. It is important to note that although the infant mortality rate has declined, the majority of under-5 deaths are neonatal. Congenital malformations are the most significant cause of neonatal death [50], but there are no studies yet to understand the environmental associations in Sri Lanka.

Outdoor air pollution has increased in Sri Lanka, and the increase is correlated to an increase in private vehicle sales [73]. Open burning of plastics emits dioxins due to lack of formal waste disposal, and it is a common contributor to outdoor air pollution. Estimates have noted that indoor air pollution remains a larger threat than outdoor air pollution in Sri Lanka (as of 2014), however data specific to Sri Lanka is limited. Nearly 66% of the population uses biomass fuel for indoor cooking in Sri Lanka [74,75]. The highest use of biomass is in the estate sector (80%), followed by the rural sector where an estimated 74% of the population uses firewood. Poor ventilation, the absence of chimneys, and the practice of using polythene (plastic bags, etc) to initiate a fire all contribute to indoor air pollution. Unlike other countries in the region, heating is rarely a contributor to indoor air pollution due to the warm natural climate. A World Bank study in Sri Lanka found that indoor air pollution was a predictor of diabetes among adults and is a predictor of stunting, underweight, and wasting in children under five [76].

Breastfeeding rates are excellent, with 90% of mothers breastfeeding for six months. Almost all deliveries (>97%) are institutional. More than 10% of the population has diabetes, and the proportion is expected to increase [77]. A recent study found that 38% of children aged between 10–14 years were obese, and 20% were overweight [78]. Increasing numbers of children are being exposed to dietary factors, sedentary behaviors, and unhealthy habits. There remains, however, a double burden of disease associated with nutritional problems in Sri Lanka because both malnutrition and obesity are increasing [79,80].

There are gaps in industries in terms of waste and chemicals disposal. Inappropriate use of agricultural chemicals is common. More than 80% of agricultural workers in the country work in the informal sector and there is limited to no personal protective equipment use while synthetic pesticide usage, particularly herbicides, is increasing [81]. CKDu is a major problem that may be related to pesticide and chemical use [82].

Dengue remains a major problem; 30% of dengue patients in 2017 were 5–19 years old. In 2017, there were more than four times the number of cases compared to the 2010 and 2016 average.

The country has seen an increase in floods, droughts, and landslides, and children and families are often forced to move due to these events.

Improved monitoring and surveillance are needed to capture baseline data, as is increased research on the environment and children’s health, particularly understanding of early exposures on adult health.

## Summary and Conclusions

Children contribute substantially to the burden of disease in countries in South and Southeast Asia, as shown in Table 1. Taken as plausibly representative of the current status of CEH in these regions, the country reports summarized in this paper show a high degree of similarity. Most common are problems with outdoor and household air pollution, with solutions not immediately apparent or implementable. Children are also often exposed to heavy metals, industrial chemicals, and pesticides. Despite advances in some countries, many children still do not have adequate access to clean water and improved sanitation. Infectious diseases remain a problem, especially for children living in poverty. Child labour is still widely prevalent (too common), generally without adequate training or personal protective equipment, exposing the child labourers to occupational hazards. The children of these regions are now facing the dual problems of undernutrition and stunting on the one hand, and overnutrition and obesity on the other.

In conclusion, it is evident that some countries in these regions are doing better than others in varying areas of CEH. By establishing and participating in regional networks, countries can learn from each other and harmonise their efforts to protect CEH so that all can benefit from closer interactions.
